# Matrix independent and interference free *in situ* boron isotope analysis by laser ablation MC-ICP-MS/MS†

**DOI:** 10.1039/d5ja00028a

**Published:** 2025-04-03

**Authors:** Christopher D. Standish, J. Andy Milton, Rachel M. Brown, Gavin L. Foster

**Affiliations:** a School of Ocean & Earth Sciences, University of Southampton, National Oceanography Centre European Way Southampton SO14 3ZH UK c.d.standish@soton.ac.uk; b Aix Marseille Université, CNRS, IRD, INRAE, Coll France, CEREGE Aix-en-Provence France

## Abstract

The accuracy of boron isotope analysis by laser ablation multi-collector inductively coupled plasma mass spectrometry (LA-MC-ICP-MS), particularly when the mass bias correction utilises non-matrix-matched reference materials, is compromised by matrix- and mass-load induced biases and an interference from scattered Ca and Ar ions which can induce bias in excess of 20‰. Here we explore the first application to *in situ* boron isotope analysis of the Thermo Scientific Neoma MS/MS mass spectrometer, which combines a traditional MC-ICP mass spectrometer with a collision/reaction cell and pre-cell mass filtering technology. While operating in full transmission mode, *i.e.* without using the collision/reaction cell, the pre-cell mass filter successfully eradicates the interference from scattered ions seen on some pre-existing models of MC-ICP-MS and exhibits good analytical sensitivity (∼6–14 mV per μg per g of total boron is typically achieved here). Furthermore, when matching laser operating parameters for samples/secondary reference materials and bracketing reference materials, and limiting the mass of ablated material introduced to the plasma, matrix- and mass-load induced biases can be prevented without the need for instrument tune conditions that severely limit sensitivity. Mean values of 14 reference materials, varying in bulk chemical composition (carbonates and silicates) and boron concentration (c. 2–150 μg g^−1^), are within uncertainty of reference values when instrumental mass bias is normalised using bracketing analyses of NIST SRM612 glass, demonstrating the accuracy and utility of this approach. Internal precision and external reproducibility are primarily controlled by boron signal intensity and both are typically better than 1‰ when the ^11^B intensity is at least ∼40 mV. LA-MC-ICP-MS/MS therefore offers a new and exciting opportunity for accurate and precise matrix independent, *in situ*, boron isotope analysis of geological materials.

## Abstract 2

LA-MC-ICP-MS/MS offers a new and exciting opportunity for accurate and precise (typically better than 1‰ when the ^11^B intensity is at least ∼40 mV) matrix independent, *in situ*, boron isotope analysis of geological materials.

## Graphical abstract

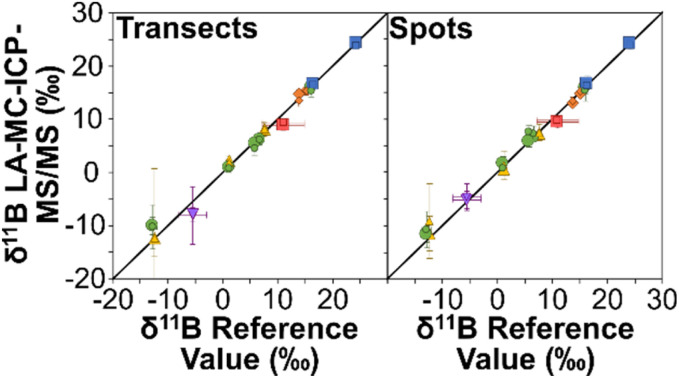

## 1 Introduction

Boron isotope analysis is a key geochemical approach used throughout the Earth and Planetary sciences,^1^ playing an important role in a diverse range of studies including those concerned with ocean acidification,^2,3^ past variations in atmospheric CO_2_,^4,5^ biomineralisation of marine calcifiers,^6,7^ mixing processes in the ocean-crust–mantle system,^8,9^ and cosmochemistry.^10,11^ Based on natural variations in the relative abundance of the two stable isotopes of boron, ^10^B (∼20%) and ^11^B (∼80%), data are typically expressed in delta notation:1
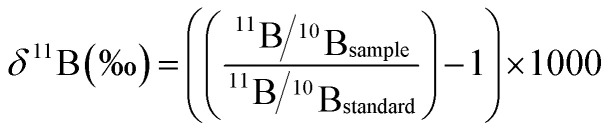
where ^11^B/^10^B_standard_ is the boron isotopic composition of the National Institute of Standards and Technology (NIST) Standard Reference Material (SRM) 951 boric acid characterised by ^11^B/^10^B of 4.04367.^12^

The most precise methods for analysing the boron isotope composition of bulk samples are thermal ionisation mass spectrometry (TIMS) and solution MC-ICP-MS, where internal precisions of <0.3‰ (2*σ*) have been achieved.^13^ However, such approaches are time consuming due to the sample pre-treatment procedures required, and sample size requirements (1–1000 ng and 2–50 ng B, respectively) severely limits their ability to characterise isotopic heterogeneities at the sub-mm scale. This restricts their effectiveness for certain applications where accurate and precise determination of μm-scale structures are required, *e.g.* in understanding differences in coral biomineralisation between different skeletal components,^7,14^ or the origin of fluids during mantle serpentinisation.^15,16^

For a number of decades, such high-resolution analyses have been possible by secondary ion mass spectrometry (SIMS),^17–19^ where spatial resolutions, dependent on the required signal intensity and the boron concentration in the sample, are on the order of ∼5–100 μm. This approach has achieved internal precisions (2SE) of 0.5–3‰ and external reproducibilities (2 SD) of 0.4–4.7‰.^13^*In situ* boron isotope analyses by LA-MC-ICP-MS has recently become more common,^6,20–22^ a technique that can analyse at similar spatial resolutions with the added advantage that 2D images can be produced facilitating correlative imaging approaches.^7,23^ Internal precisions of ∼0.5‰ (2 SE) for carbonates^24,25^ and ∼0.2‰ (2 SE) for tourmalines,^26,27^ alongside external reproducibilities of ≤1.0‰ (2 SD) for both materials^24,25,27,28^ as well as silicate glasses,^29,30^ have all been achieved with this technique. However, whilst accuracy of ≤1.0‰ has been demonstrated in numerous studies dealing with the aforementioned matrices,^26,29–32^ it has proven to be problematic in others. This has primarily been when analysing carbonates without matrix-matched reference materials, where inaccuracies up to and in excess of 20‰ have been reported,^24,33,34^ although inaccuracies of up to 2.5‰ have also been reported when analysing silicate minerals.^27^

Difficulties achieving accurate boron isotope measurements by LA-MC-ICP-MS appear to relate to two key processes. Firstly, matrix- and mass-load induced biases of up to several permil have been identified in a number of studies.^25,30,34–36^ With respect to ablation rate, an important factor if non-matrix matched reference materials are used, Coenen *et al.*^36^ estimated that the boron isotope bias would be −0.27 ± 0.22‰ when the standard/analyte ablation ratio varies over a range of 1 to ∼15, and although this is not resolvable in the context of typical internal precision or external reproducibility, it could result in long-term inaccuracy.

Fietzke and Anagnostou^37^ relate matrix- and mass-load induced biases to plasma conditions,^38^ with inaccuracy induced if contrasting sample matrices behave differently in terms of atomisation, ionisation, and mass fractionation within the plasma. Using an AXIOM MC-ICP mass spectrometer in multi-ion counting mode, they demonstrated that the *δ*^11^B composition of non-matrix matched reference materials are inaccurate by up to ∼3‰ when analysed under “cold plasma” conditions (higher Ar sample gas flows, higher sensitivity, highest rate of change in isotopic fractionation with changing plasma conditions). In contrast, under “hot plasma” conditions where better matrix tolerance is typically achieved (lower Ar sample gas flows, significantly lower sensitivity, lowest rate of change in isotopic fractionation with changing plasma conditions), the *δ*^11^B composition of non-matrix matched reference materials agree with published values. Furthermore, greater degrees of isotopic fractionation when skimmer cones were heavily coated in ablated material indicated that fractionation within the ICP interface is also likely an issue.^37^

**Fig. 1:**
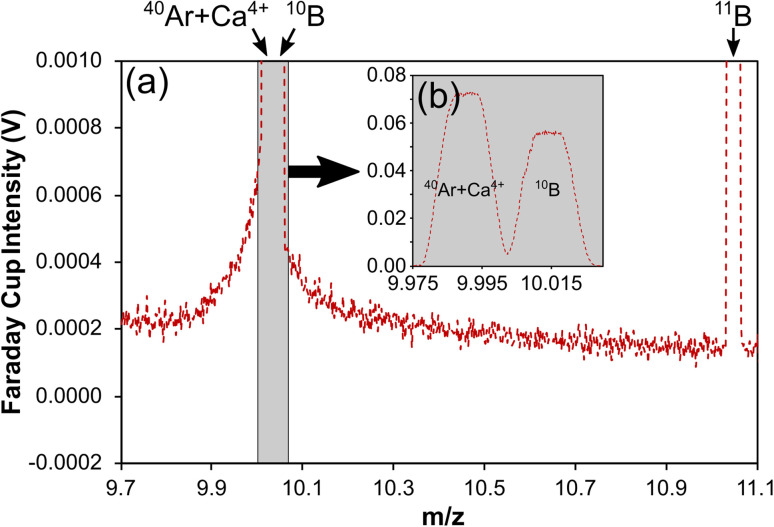
(a) Mass scan across the boron isotope mass range when ablating a carbonate reference material collected on a Neptune MC-ICP mass spectrometer,^24^ showing the elevated baseline across the entire boron mass range that acts as an interference and impacts the accuracy of boron isotope measurements by LA-MC-ICP-MS. (b) A mass scan showing the well-resolved ^40^Ar + Ca^4+^ and ^10^B peaks.

Despite the quadruply charged mass 40 peak (*m*/*z* ∼9.99), which consists of both Ar^4+^ and Ca^4+^ ions, sitting to the low-mass side of the ^10^B peak, this is well resolved in most available mass spectrometers so that it does not pose an isobaric interference in itself (Fig. 1). Instead, the second issue resulting in *δ*^11^B bias relates to an interference across the entire boron mass range that results from scattered ions, which can induce inaccuracies in excess of 20‰.^24,25,34,36,37^ Identified first on the Thermo Scientific Neptune Plus when ion detection employed Faraday cups, the scattered ions manifest as an elevated and irregular baseline that peaks in intensity around the mass region of ^10^B and the quadruply charged mass 40 peaks (Fig. 1).^24,34^ A similar elevated baseline has since been shown to exist on the Thermo Scientific AXIOM and the Nu Instruments Plasma 3 also when using Faraday cup detectors,^37^ although the shape of the baselines do differ between models and thus the severity of this interference is likely to differ also. Inaccuracy occurs because instrumental mass bias correction of raw ^11^B/^10^B is typically achieved by standard-sample bracketing, and the proportion of interference to analyte-derived signal on the boron isotope masses differs between samples/secondary reference materials and the normalising reference material if the boron and/or the interference signal intensities differ.

Such baseline interferences are not apparent when detection employs ion counters with pre-detector deflection (*i.e.* for off-axis detectors) or filtration.^24,37^ Although some studies attribute accuracy to the use of femto-second rather than nano-second laser ablation systems,^32,39^ these studies also employ ion counters for detection and thus the reason for the apparent accuracy in these instances remains an open question. Such detector-related observations support the hypothesis that scattered ions are the principal cause of this elevated baseline around the boron masses because pre-detector deflection of the focussed ion beam and/or energy filtration to remove scattered ions with disturbed energies would prevent the scattered ions from reaching the instrument's detectors.^24,37^

Standish *et al.*^24^ used laser ablation analysis to demonstrate how the baseline intensity scales with [Ca], whilst Coenen *et al.*^36^ explored the source of this baseline interference during analysis of Ca-containing solutions and documented a baseline that was ∼4 and ∼14 times higher around *m*/*z* ≈ 10 when under wet and dry plasma conditions respectively. Both observations indicate that Ca ions are a key component of this elevated baseline. Coenen *et al.*^36^ found no baseline elevation under the presence of a Mg matrix, thus indicating that sample matrix elements beyond Ca are unlikely to be the cause. Clearly this elevated baseline represents an analytical challenge when targeting calcium carbonate materials due to their high Ca content (∼40 wt%), however this problem is not confined only to such materials. The inaccuracy documented by Míková *et al.*^27^ for tourmalines analysed by LA-MC-ICP-MS can also be explained by differing Ca contents,^24^ whilst Evans *et al.*^25^ demonstrated the Ca-based interference is linked to inaccuracy in other silicates. Normalisation is typically achieved through the analyses of NIST glass reference materials such as SRM610 or SRM612, that have [Ca] on the order of ∼8 wt%. It is therefore likely that this will induce some degree of inaccuracy in any material with a higher or lower Ca content, or indeed contrasting B/Ca.

Based on a linear response of the baseline around *m*/*z* 10 to ^38^Ar intensity when altering plasma conditions, Fietzke and Anagnostou^37^ proposed that as well as Ca ions, scattered Ar ions are also responsible for the elevated background in the boron isotope mass range. This explains the elevated background documented during blank measurements.^24,25,34^ It also explains the observation made by Coenen *et al.*^36^ of elevated baseline scans during ablation of (Ca-free) magnesite compared to blank (laser-off) because the ^40^Ar^4+^ ion beam intensity is also elevated during ablation relative to the blank measurement. Coenen *et al.*^36^ ablated a series of reference materials with different [Ca], and whilst a significant linear relationship was found when regressing the intensity of the interference around *m*/*z* ≈ 10 against analyte matrix [Ca], a more robust relationship was found when regressing the interference against analyte matrix [Ca] and the ^40^Ar^4+^ ion beam intensity. This confirmed that Ca and Ar ions together contribute to the elevated baseline in the boron isotope mass range, and as noted by Fietzke and Anagnostou,^37^ it is likely that ^40^Ar and ^40^Ca are the main cause because both elements have a 40 amu isotope and in both cases it is the element's most abundant isotope (99.6% and 96.9%, respectively).

To counter this baseline interference, numerous studies have developed interference correction protocols to enable accurate boron isotope analysis to be achieved. Sadekov *et al.*^34^ adopted an off-peak baseline correction method, subtracting baseline intensities measured immediately to the side of the boron isotope peaks from the intensities of the boron isotope peaks. Standish *et al.*^24^ and Evans *et al.*^25^ analysed a suite of reference materials with differing B/Ca to characterise the well-defined relationship between boron isotope inaccuracy (Δ*δ*^11^B) and ^11^B/interference, where the interference is measured immediately to the high-mass side of the ^10^B peak, in order to apply a baseline interference correction. Both correction protocols are therefore based on the ratio of boron intensity to interference intensity for each individual measurement, and not simply on their B/Ca ratio;^37^ a subtle but important difference if both Ca and Ar are the cause.

The latter approach in particular has been shown to produce accurate measurements in a number of studies,^7,21–23,40^ thus whilst successfully correcting for the baseline interference it must also correct for any other plasma induced matrix- and mass-load biases; *e.g.* long-term collation of deep-sea coral reference material PS69/318-1 from the aforementioned papers gives a mean (±2SD) value of 14.07 ± 0.76‰, close to the solution value of 13.83 ± 0.29‰ respectively. Following that this approach includes tuning of the mass spectrometer for maximum sensitivity, it also means that it is possible to achieve maximum sensitivity and accuracy at the same time, albeit with the help of secondary normalisation to matrix-matched reference materials. Nonetheless, the ability to analyse the boron isotope composition of a range of solid materials by LA-MC-ICP-MS without both matrix- and mass-load induced biases or the need for additional baseline corrections is highly desirable, due to the potential for better detection limits, improved accuracy, lower uncertainty, and faster sample through-put. Alternative approaches are therefore of great interest to the discipline.

Recent commercialisation of a new type of MC-ICP mass spectrometer, the Thermo Scientific Neoma MS/MS mass spectrometer, combines a conventional MC-ICP mass spectrometer (an updated design compared to its predecessor the Neptune MC-ICP mass spectrometer) with a collision/reaction cell and pre-cell mass filtering technology that is located between the ICP interface and the electro-static analyser (ESA).^41–44^ The pre-cell mass filter consists of a double-Wein filter that permits transmission of a chosen mass range using a combination of electrostatic fields (*E*), magnetic fields (*B*), and an adjustable slit aperture; the combination of *E* and *B* on the first Wein filter deflects ions away from the axial trajectory, the adjustable slit aperture transmits ions within a certain mass range, and the *E* and *B* of the second Wein filter refocuses the ions to the axial trajectory prior to further beam refocussing by several lenses.^42^ Collision/reaction cells enable the reduction or removal of isobaric interferences on targeted masses by either the selective chemical reaction of an analyte or interfering species with a gas to induce a mass shift, or preferential collision of a polyatomic interference with a gas in order to dissociate their chemical components.^45^ The pre-cell mass filter can be used even when the collision/reaction cell is not utilised (known as “full transmission mode”), enabling removal of matrix elements and plasma gases which can boost sensitivity of the target masses.^46^ Here we utilise the Thermo Scientific Neoma MS/MS MC-ICP mass spectrometer in full transmission mode to: (1) explore the potential of prefilter technology to remove spectral baseline interferences, and (2) assess its potential for accurate and precise determination of boron isotope ratios when coupled to a nanosecond laser ablation system.

## 2 Materials and methods

### 2.1 Reference materials

All reference materials analysed as part of this study are listed in Table 1. They have either previously been characterised for their boron concentration and isotopic composition using standard analytical approaches, such as solution ICP-MS, TIMS, or SIMS, or have been characterised by solution ICP-MS as part of the present study.

**Table 1:** Reference materials used in this study

ID	Description	B	B/Ca	2*σ*	*δ* ^11^B	2*σ*	Ref.
		(μg g^−1^)	(μmol mol^−1^)		(‰)		
NIST610	Silicate glass	356.0	16 198.2	—	−0.26	0.13	24 and 47
UB-N-μp	Micropowder pellet of peridotite from the Vosges mountains, France	148.3	64 369.7	13 977.8	11.0	3.8	47
JCp-1-μp	Micropowder pellet of *Porites* sp. coral (JGS)	49.7	459.6	45.4	24.4	0.5	48 and 49
DE-B-c	Inorganic blue calcite rhomb	48.2	446.6	14.8	1.2	0.3	This study
DE-B-np	Nanopowder pellet of inorganic blue calcite rhomb	41.3	382.9	13.3	1.1	0.2	This study
NIST612	Silicate glass	35.0	1525.6	—	−0.5	—	25 and 47
PS69/318-1b	Cold water calcitic scleraxonian octocoral	25.2	233.8	44.8	15.3	0.3	7
PS69/318-1	Cold water calcitic scleraxonian octocoral	21.4	198.0	7.9	13.8	0.3	24 and 50
JCt-1-μp	Micropowder pellet of aragonite, *Tridacna gigas* (JGS)	20.6	191.0	19	16.4	0.6	48 and 49
NF10-np	Nanopowder of aragonitic *Heliopora* sp. octocoral	17.1	158.7	8.2	16	0.17	This study
UWC-1-np	Nanopowder pellet of inorganic calcite rhomb, Valentine Wollastonite Mine	13.4	123.8	3.0	6.7	0.5	This study
UWC-1-c	Inorganic calcite rhomb, Valentine Wollastonite Mine	13.2	122.5	9.8	7.6	0.5	This study
BCR-2G	Basalt glass	9.4	694.3	427.0	−5.5	2.6	47
UWC50-np	Nanopowder pellet of inorganic calcite rhomb, Valentine Wollastonite Mine, mixed 1 : 1 with ∼B-free carbonate powder	7.1	66.0	1.8	5.8	0.4	This study
DE-Y-c	Inorganic yellow calcite rhomb	1.8	16.4	0.9	−12.8	0.9	This study
DE-Y-np	Nanopowder pellet of inorganic yellow calcite rhomb	1.8	17.1	2.7	−12.4	0.2	This study

Powdered reference materials JCp-1, a *Porites* sp. coral,^51^ and JCt-1, a biogenic aragonite *Tridacna gigas*^52^ are well characterised, having been the focus of interlaboratory comparison studies by Gutjahr *et al.*^49^ for *δ*^11^B and Hathorne *et al.*^48^ for B/Ca. Cold water calcitic scleraxonian octocoral PS69/318-1 and PS69/318-1b are fragments of the same coral specimen, but with slight differences in boron geochemistry.^7,24,50^ NIST SRM612 (silicate glass), UB-N (powdered peridotite from the Vosges mountains, France), and BCR-2G (basaltic glass) have been the subject of a number of studies, and here we use mean values derived from the GeoReM database.^47^ NF10 is a specimen of an aragonitic *Heliopora* sp. octocoral whilst UWC-1, DE-B, and DE-Y are specimens of calcite acquired from mineral dealerships. All have been characterised as part of this study (ESI Table S1†). UWC-1 is sourced from the Valentine Wollastonite Mine in the Adirondacks, USA,^53^ and has been previously measured for *δ*^11^B by Foster *et al.*^50^ and Standish *et al.*^24^ using solution MC-ICP-MS (mean ± 2SD of +8.35 ± 0.45‰ and +7.77 ± 0.89‰ respectively), and by Kasemann *et al.*^17^ using SIMS, TIMS, and solution MC-ICP-MS (mean ± 2 SD of +8.08 ± 1.16‰). Coenen *et al.*^54^ report a mean (±2 SD) solution MC-ICP-MS *δ*^11^B value of −0.02 ± 0.41‰ for DE-B, albeit this is based on analyses of a different hand specimen. DE-Y^36^ has not previously been characterised for its boron isotope composition.

Calcites were analysed in the form of both calcite rhombs (suffix “c”) and as pressed nanopowder pellets (suffix “np”), whilst NF10 was analysed as a pressed nanopowder pellet only. An additional pressed nanopowder pellet reference material, UWC50, is comprised of UWC-1 mixed with a low boron (c. 0.1 μg per g B) calcium carbonate powder (Thermo Scientific Alfa Aesar, LOT: Q09G066, c. 0.1 μg per g B) at a ratio of 1 : 1. Fragments of glass, coral, and calcite were mounted in MetPrep EPO-FLO high purity, low viscosity, clear epoxy resin, then were polished using 2500 grit paper and cleaned with isopropanol alcohol prior to analysis. Micro- (suffix “μp”) and nanopowder pellets were mounted in custom-made epoxy mounts. Details of their production follows below.

### 2.2 Pressed powder pellets

For the creation of nanopowder, carbonates (calcites) were first crushed in a jaw-crusher to a particle size of ≤2 mm, and then the resulting particles were milled in a Fritsch planetary ball mill at 600 Hz using 45 ml agate vials and 32 g of 5 mm agate balls. Milling was performed on aliquots of 1.5 g of carbonate with 8 g of 18.2 MΩ cm (ultrapure) water and consisted of 10× 3-minute cycles at 900 rpm with 2-minute pauses between each cycle. For BE-B-np, DE-Y-np, and UWC-1-np, the reference material accounts for 100% of the 1.5 g of carbonate milled. For UWC50-np, 0.75 g of UWC-1 was milled will 0.75 g of the aforementioned calcium carbonate powder to result in a nanopowder with half the boron content of UWC-1. Following milling, the slurry was dried at c. 50 °C overnight, re-homogenised using an agate hand pestle and mortar, then stored in acid-cleaned glass vials. Between each use, the pestle and mortar were ultrasonicated in ultrapure water for 5 minutes, then rinsed 3 times in ultrapure water, wiped with isopropanol alcohol, and dried using compressed nitrogen gas. Prior to milling a new reference material, the mill was cleaned/conditioned by twice completing the full milling procedure using either the aforementioned low-boron calcium carbonate powder, or the reference material in question. The resulting slurry was disposed of each time.

Pressed powder pellets, 10 mm in diameter, were produced using a manual hydraulic press (Specac, Orpington, UK) and a stainless-steel pellet die. For micropowder pellets (UB-N-μp, JCp-1-μp, JCt-1-μp), c. 120 mg of powder was pressed at 5 MPa for 5 minutes. For nanopowder pellets (DE-B-np, NF10-np, UWC-1-np, UWC50-np, DE-Y-np), c. 80 mg of powder was pressed on top of 150 mg of 20 μm microcrystalline cellulose powder (PN310697, SigmaAldrich) at 5 MPa for 5 minutes. The cellulose backing was used so that nanopowder pellets can be produced using less reference material. Between presses of different materials, the pellet die was cleaned by ultrasonication in ultrapure water for 5 minutes followed by 3× rinses in ultrapure water, wiped with isopropanol alcohol, then dried using compressed nitrogen gas.

### 2.3 Solution ICP-MS and MC-ICP-MS

All calcite crystal and nanopowder reference materials used in this study were characterised for their geochemical composition (element/calcium ratios, *δ*^11^B) by solution ICP-MS. Results are provided in ESI Table S1.† Crystals or powders (∼1 mg) were dissolved in ∼0.15 M HNO_3_ and separated into two fractions: ∼10% for elemental analysis and ∼90% for isotopic analysis. The trace element fractions were diluted and measured on an Element XR ICP mass spectrometer (Thermo Fisher Scientific, Waltham, MA, USA) following Henehan *et al.*^55^ Analytical reproducibility is better than ± 5% (2 SD) for Li/Ca, B/Ca, Na/Ca, Mg/Ca, Mn/Ca, Sr/Ca, Cd/Ca, Ba/Ca, Nd/Ca, and U/Ca, and better than ± 30% (2 SD) for Al/Ca and Fe/Ca. Boron was separated from the matrix of the isotope fraction with anion exchange resin Amberlite IRA743 *via* a batch purification method.^56^ Samples were processed alongside secondary reference material NIST SRM8301 Foram, a synthetic foraminifera solution,^57^ and total procedural blanks (TPB). Isotopic analysis was performed on a Neptune Plus MC-ICP mass spectrometer (Thermo Fisher Scientific), with instrumental mass bias corrected by standard-sample bracketing with NIST SRM951, following Foster^58^ and Foster *et al.*^50^ All TPBs were <0.1% of the sample size and hence negligible. The solution concentrations ranged from 10 to 40 ppb boron. Repeat measurements of NIST SRM8301 Foram across multiple sessions gives a mean (±2SD) of 14.71‰ ± 0.23‰ (*n* = 24), consistent with the reference value of +14.51‰ ± 0.17‰.^57^

### 2.4 Laser ablation MC-ICP-MS/MS

Laser ablation boron isotope analysis was performed at the University of Southampton using a Neoma collision/reaction cell MC-ICP mass spectrometer equipped with 11 faraday cup detectors and a central ion counter, coupled to an Elemental Scientific Lasers (Bozeman, MT, USA) NWR193 excimer laser ablation system with a TwoVol2 ablation chamber. ^10^B and ^11^B were measured on the L5 and C Faraday cups respectively, installed with 10^11^ Ω resistors, whilst ^12^C was measured on the H5 Faraday cup installed with a 10^10^ Ω resistor. Instrumental tuning protocols, implemented whilst ablating silicate glass NIST SRM610, were designed to balance high sensitivity with high ^11^B/^10^B stability. Maximum stability is typically achieved by adjusting the argon make-up gas, resulting in a 5–10% loss in boron intensity from peak sensitivity. Tuning was performed by ablating NIST SRM610 with a square 100 by 100 μm diameter laser beam at 5 Hz repetition rate and with a laser energy density of ∼4.5 J cm^−2^, typically achieving a sensitivity of 1.6 V on ^11^B. This equates to ∼6 mV per μg per g B.

Potential surface contamination was removed prior to data collection in transect mode by ablating the sample and standard surfaces with a low laser energy density (∼0.8 J cm^−2^) and fast laser tracking speed and repetition rate (400 μm s^−1^ and 40 Hz, respectively). Operating conditions during data acquisition are detailed in Table 2. Measurements of reference materials consisted either of 120 s of data collection when the laser ablation system was operating in transect mode (transect length c. 1.2 mm), or 45 s of data collection when the laser ablation system was operating in spot mode, with gas blanks characterised over the 40 s immediately preceding and succeeding each measurement. A variety of laser ablation parameters were explored (Table 2). Data reduction was performed in iolite 4 (ref. 59) using a custom-made data reduction scheme (DRS) “B_Neoma” (ESI†). Following a 2 s trim at the start and end of the ablation to account for instability in the signal as the ablation both starts and finishes, on-peak blank corrections were applied to each measured mass cycle by cycle following smoothing of the blank intensities using a spline function (“LinearFit”). Instrumental mass bias was corrected by standard-sample bracketing with NIST SRM612 glass reference material using a spline function (“LinearFit” spline) and the isotope composition stated in Table 1 (−0.5‰).

Operating conditions for laser ablation MC-ICP-MS/MS

**Table:** 

Mass spectrometry	
Mass spectrometer	Thermo Scientific Neoma collision/reaction cell multi-collector inductively coupled plasma mass spectrometer
Cones	Nickel skimmer (X) and nickel Jet sample
RF power	1200 W
Cooling gas (argon)	14 l min^−1^
Auxiliary gas (argon)	0.8 l min^−1^
Make-up gas (argon)	1.030 to 1.075 l min^−1^
Wien magnetic field	17%
Wien electrostatic field	262.0 V
Wien focus 1 base	−440.0 V
Wien focus 1 *X*-symmetry	10.0 V
Wien focus 1 *Y*-symmetry	17.0 V
Slit aperture	70%
Wien focus 2 base	−429.5 V
Wien focus 3 base	−410.0 V
Wien focus 3 *X*-symmetry	−4.0 V
Wien focus 3 *Y*-symmetry	−26.0 V
CCT entry	−36.0 V
CCT bias	0.0 V
CCT RF amplitude	30%
CCT exit 1	−130.0 V
CCT exit 2	−100.0 V

**Table:** 

Laser ablation system	
Laser ablation system	Elemental scientific lasers NWR193 excimer laser ablation system with a TwoVol2 ablation chamber
Ablation cell carrier gas (helium)	0.45 to 0.50 l min^−1^
Additional gas (nitrogen)	0.004 to 0.008 l min^−1^
Laser energy density	∼1 to 4.5 J cm^−2^
Laser repetition rate	5 to 40 Hz
Laser beam size	50 by 50 μm to 100 by 100 μm squares; 50 μm to 80 μm diameter circles
Laser tracking speed	10 to 400 μm s^−1^
Ablation mode	Transect and spot

## 3 Results and discussion

### 3.1 Accuracy, internal precision, and external reproducibility

To assess the general capabilities LA-MC-ICP-MS/MS for *in situ* boron isotope analysis, 14 reference materials selected to cover a range of bulk chemical compositions (silicates and carbonates), physical matrices (pressed powdered pellets, crystals, glass), boron concentrations (c. 2–150 μg g^−1^), and B/Ca ratios (c. 16–64,000 μmol mol^−1^), have been analysed. Bracketing reference materials (NIST SRM612) were always ablated with the same laser parameters as the secondary reference materials that were under investigation. Four separate analytical set-ups were employed: two with the laser operating in transect mode using a 50 by 50 μm or 80 by 80 μm square laser beam (whilst tracking at 10 μm s^−1^); and two with the laser operating in spot mode with a 50 μm diameter or an 80 μm diameter circular laser beam. All four analytical set-ups employed a laser repetition rate of 12 Hz and laser energy density of ∼4.5 J cm^−2^, with further details available in the Materials and methods. A minimum of 5 analyses were performed on each reference material per analytical set-up, with each analytical sequence lasting for no more than 6 hours. Sensitivities are typically in the range of ∼6–14 mV per μg per g boron (*i.e.*^10^B and ^11^B signals combined) for both spots and transects when using the larger laser beam sizes.

**Fig. 2:**
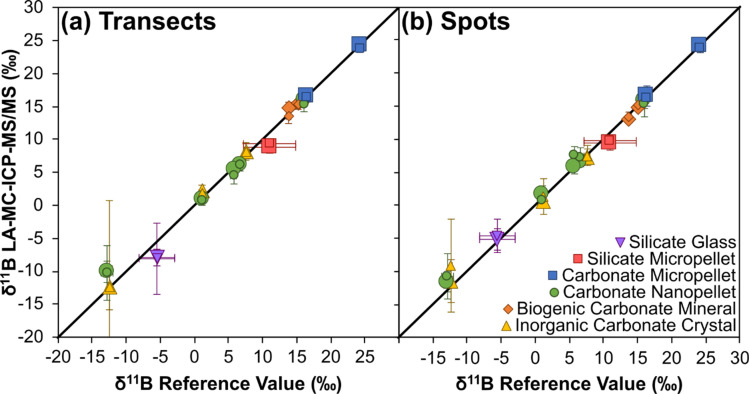
Mean LA-MC-ICP-MS/MS *δ*^11^B (± 2SD) *versus* reference value *δ*^11^B (Table 1) for the 14 reference materials analysed in this study: (a) transect measurements, (b) spot measurements. Data label size correlates to laser beam size: smaller labels correspond to either 50 by 50 μm square (transect) or 50 μm diameter circle (spot) laser beams, and larger labels correspond to 80 by 80 μm square (transect) or 80 μm diameter circle (spot) laser beams. Solid black line represents 1 : 1 ratio.

**Fig. 3:**
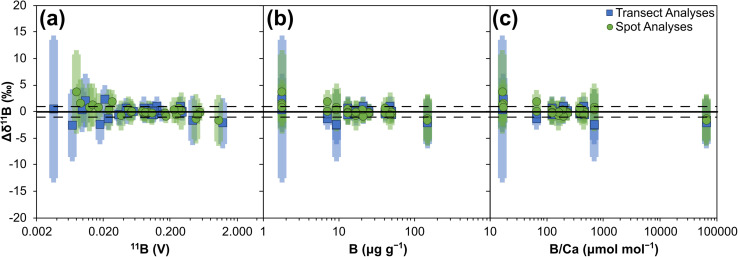
Boron isotope accuracy (Δ*δ*^11^B) of 14 reference materials analysed by LA-MC-ICP-MS/MS: (a) plotted against ^11^B signal (V), (b) plotted against boron μg g^−1^, and (c) plotted against B/Ca (μmol mol^−1^). Solid horizontal bar represents Δ*δ*^11^B of 0‰, dashed horizontal bar represents Δ*δ*^11^B of +1‰ and −1‰ respectively. Uncertainties are the 2SD of the repeat measurements by LA-MC-ICP-MS/MS summed in quadrature with the uncertainties of the reference values presented in Table 1.

Data are provided in ESI Tables S2–S5.† Mean values of all reference materials are within uncertainty (2 SD) of their reference values, with accuracy achieved on all matrix types, *i.e.* micro-pellet, nano-pellet, biogenic crystals and inorganic crystals (Fig. 2). Furthermore, no strong correlations between accuracy and either boron signal, boron concentration, or B/Ca exist (Fig. 3), with Pearson's correlation coefficients all <0.5. These results therefore indicate that accurate, *in situ*, matrix independent boron isotope analysis is achievable by LA-MC-ICP-MS/MS. Importantly, this is accomplished with ion detection employing Faraday cup detectors rather than ion counters, thus reaping the benefits of measuring higher and more stable signals.^60^ Furthermore, specialist tuning protocols, for example those that tune for plasma conditions to negate matrix and/or mass-load induced biases,^38^ are not required to achieve accuracy.

**Fig. 4:**
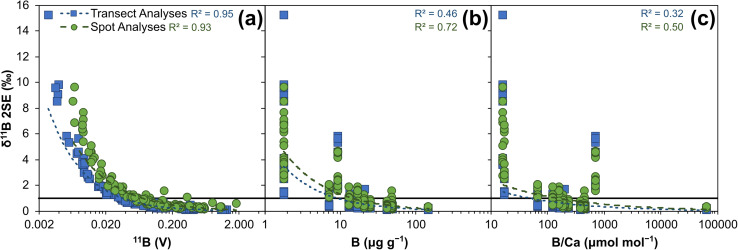
Boron isotope internal precision (2 SE) of 14 reference materials analysed by LA-MC-ICP-MS/MS: (a) plotted against ^11^B signal (V), (b) plotted against boron μg g^−1^, and (c) plotted against B/Ca (μmol mol^−1^). Solid black line represents 2 SE of 1‰. Dashed coloured lines show power relationship of all transect or sport data respectively, with *R*^2^ included.

Internal precision, expressed as ±2 standard errors (SE) of the mean of the total number of integration cycles, are primarily controlled by boron signal intensity and range from ∼0.1‰ at higher (∼1.3 V ^11^B) boron signal intensities to ∼15.2‰ at lower (∼0.003 V ^11^B) boron signal intensities (Fig. 4). Internal precisions are better than 1‰ when the intensity of ^11^B is at least ∼40 mV (Fig. 4a). The difference in the 2SE for spot *versus* transect measurements visible in Fig. 3a relates to counting statistics, with spot measurements comprised of fewer integrations (∼40 compared to ∼115 integrations). Relationships between internal precision and both boron concentration and B/Ca are not as strong as with boron intensity, likely due to the variable ablation efficiencies of the different reference materials studied.^36^ It is likely that precision of low intensity beams will be improved if Faraday cups utilise amplifiers with 10^13^ Ω resistors, instead of the 10^11^ Ω resistors employed here.

**Fig. 5:**
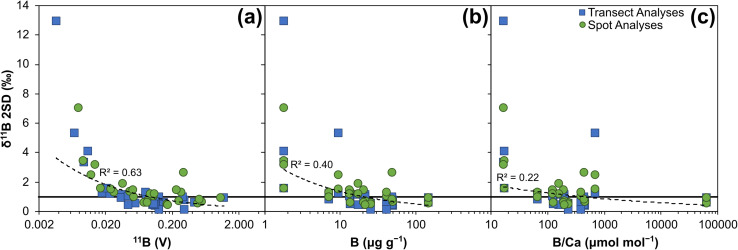
Boron isotope external reproducibility (2SD) of 14 reference materials analysed by LA-MC-ICP-MS/MS: (a) plotted against ^11^B signal (V), (b) plotted against boron μg g^−1^, and (c) plotted against B/Ca (μmol mol^−1^). Solid black line represents 2SD of 1‰. Dashed black line shows power relationship of all data, with *R*^2^ included.

External reproducibility, expressed as ±2 standard deviations (SD) of the mean of the total number of analyses, is primarily controlled by the boron signal intensity and is therefore largely a function of counting statistics, ranging from below ∼1‰ at higher (∼1.3 V ^11^B) boron intensities to ∼13‰ at lower (∼0.003 V ^11^B) boron intensities (Fig. 5). They are typically better than 1‰ when the intensity of ^11^B is at least ∼40 mV (Fig. 5a), and with the ablation settings used here this equates to boron concentrations of ∼10 μg g^−1^. Specific reference materials that plot away from the main trend in Fig. 5a are likely characterised by greater degrees of natural isotopic variation. As with internal precision, the relationship to both boron concentration and B/Ca are not as strong as with boron intensity, and this likely relates to the variable ablation efficiencies of the different reference materials studied.

Accurate measurement of *δ*^11^B by LA-MC-ICP-MS can be challenging when mass bias correction utilises NIST glass standard reference materials due to an interference from scattered Ca and Ar ions across the boron mass range and/or matrix- and mass-load induced biases.^24,34,37^Fig. 3 and 4 indicate that such issues are clearly overcome with our chosen laser conditions when using the Neoma MC-ICP-MS/MS. In the follow sections we will explore why *δ*^11^B accuracy can more readily be achieved using LA-MC-ICP-MS/MS.

### 3.2 Interference from scattered Ca and Ar ions

**Fig. 6:**
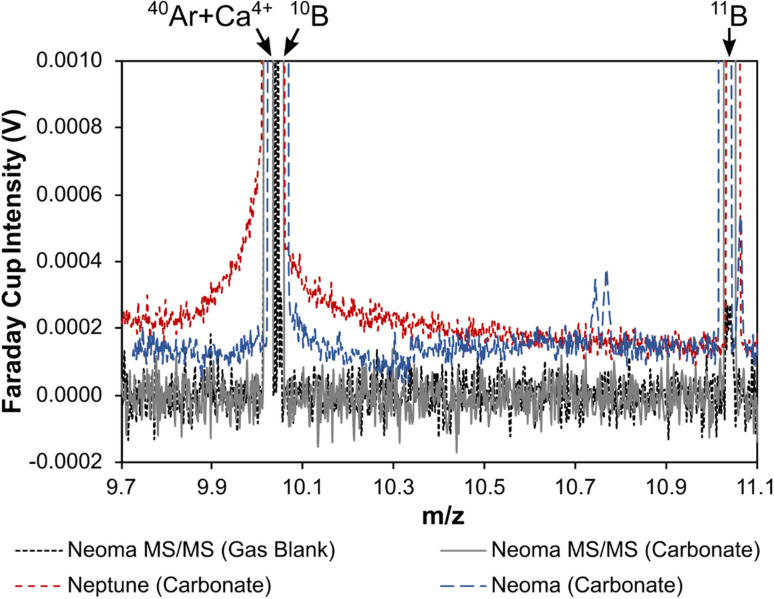
Mass scans across the boron isotope mass range when ablating carbonate reference materials collected on a Neptune MC-ICP mass spectrometer,^24^ Neoma MC-ICP mass spectrometer, and Neoma MS/MS MC-ICP mass spectrometer. A gas blank mass scan is also shown for the Neoma MS/MS MC-ICP mass spectrometer. Data from the scans are provided in ESI Table S6.†

Fig. 6 shows a series of mass scans recorded from *m*/*z* of 9.7 to *m*/*z* of 11.1 on various models of MC-ICP mass spectrometer where ion detection employed Faraday cups. The mass scan collected on a Thermo Scientific Neptune MC-ICP mass spectrometer^24^ clearly shows the irregular and elevated baseline of scattered Ca and Ar ions that has previously been linked to inaccuracies in excess of 20‰ (Fig. 6, dashed red line). In contrast, the Thermo Scientific Neoma MS/MS MC-ICP mass spectrometer (operated in full-transmission mode) scan (Fig. 6, solid grey line) shows an absence of this elevated baseline. On the basis that this baseline is thought to be one of the key causes of inaccuracy on MC-ICP mass spectrometers,^24,25,34,37^ particularly when ion detection employs Faraday cups, its absence is likely a key factor behind the accurate data presented in Section 3.1.

Due to insufficient mass resolving power, the reduction in baseline around ^10^B cannot be due to increased mass resolution of the ^40^Ar + Ca^4+^ from the ^10^B in the pre-cell mass filter. Considering this elevated baseline extends across the entire boron mass range this was unlikely to be the case, so to further investigate whether the change of baseline characteristics is related to the use of a pre-cell mass filter, or relates to other design features of the Neoma MC-ICP mass spectrometer, a scan was also collected on a base-Neoma in the Thermo Scientific Factory, Bremen, *i.e.* on a Neoma without the collision/reaction cell and pre-cell mass filter, using similar laser parameters (Fig. 6, dashed blue line; G. Craig, unpublished data). Whilst the elevated baseline is present, its intensity is lower than on a Neptune mass spectrometer. Given that some reduction in baseline occurs in the base-Neoma, the pre-filter cannot be responsible for all the reduction in scattered ions seen on the Neoma MS/MS MC-ICP mass spectrometer, and following that pre-detector deflection or filtration also removes this elevated baseline,^24,37^ one possible explanation of this reduction is that the new detector housing design of the Neoma has reduced the degree to which scattered ions are deflected into the path of the Faraday cup detectors. Other design features do differ between the base-Neoma and Neptune however, including the RF generator and torch, so the exact reason for this reduction remains unclear. Whilst it would be worthy to assess the accuracy achievable on a base-Neoma (this was not possible as part of the present study), Fig. 6 nonetheless shows that the Neoma MS/MS goes further than the base-Neoma by eradicating this baseline. This can be linked to the use of a pre-cell mass filter which both prevents Ca and Ar ions (that form the elevated baseline of scattered ions) from entering the ESA and reduces the total number of ions in the system that could cause scattering events.

**Fig. 7:**
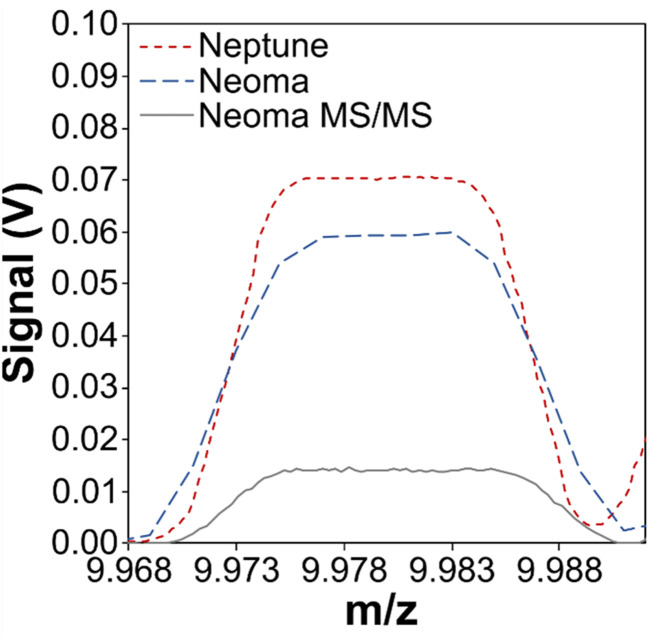
Mass scan from *m*/*z* of 9.968 to 9.992 showing the ^40^Ar + Ca^4+^ peak when ablating NIST SRM612 or JCp-1 (transect mode, 100 μm diameter laser beam, 6 J cm^−2^ laser energy density, 12 Hz repetition rate, 10 μm s^−1^ tracking speed) on a Neptune MC-ICP mass spectrometer (dashed red line), a Neoma MC-ICP mass spectrometer (dashed blue line), and a Neoma MS/MS MC-ICP mass spectrometer (solid grey line). Note that normalising to sensitivity does not impact the overall picture.

The elevated baselines shown in Fig. 6 are characterised by primary peaks (albeit broad ones) that coincide approximately with the well-resolved quadruply charged mass 40 peak, which comprises of both Ar and Ca ions (^40^Ar + Ca^4+^; Fig. 1). It has been demonstrated that the elevated baseline consists of only Ar and Ca ions, both of which have a primary isotope at mass 40 and contribute to the quadruply charged peak with *m*/*z* ∼9.99, and other matrix ions do not contribute to it.^36,37^ It therefore seems likely that the elevated baseline consists primarily or solely of ^40^Ar^4+^ and ^40^Ca^4+^ ions, and that the difference in baseline intensity between models of mass spectrometer could also be the result of differing efficiencies of generating multiply charged ions. Indeed, a similar pattern emerges with the intensities of the ^40^Ar + Ca^4+^ peaks (Fig. 7) as is described for the intensities of the baseline profiles in Fig. 6: the base Neoma is characterised by a smaller ^40^Ar + Ca^4+^ beam relative to the Neptune (a factor of ∼1.2), and the Neoma MS/MS ^40^Ar + Ca^4+^ beam is smaller again (a factor of ∼5, despite improved sensitivity over that of the Neptune by a factor of ∼1.4). With this peak primarily comprising of ^40^Ar^4+^ ions, and all instruments operating with similar make-up Ar gas flows (∼1 l min^−1^), this indicates that differences in the generation of quadruply charged ions do exist between these models of mass spectrometer, and whilst these factors are not quite as large as those seen in the reduction of the elevated baselines (factors of ∼3.8 between the Neptune and base-Neoma and ∼6.9 between the Neptune and Neoma MS/MS if measurements are taken to the side of the well-resolved ^40^Ar + Ca^4+^ peaks shown in Fig. 6), this observation is consistent with the hypothesis that the elevated baseline is, at least to some degree, comprised of scattered ^40^Ar^4+^ and ^40^Ca^4+^ ions. Changes in baseline intensity could therefore be a result of differences in the generation of multiply charged ions.

Multiply charged ions are traditionally thought to form exclusively in the ICP source.^61,62^ Differences in the generation of multiply charged ions between the Neptune and base-Neoma may therefore be explained by differences in the physical characteristics of the ICP that each instrument generates; components that combine to generate the ICP are different between the two models, including the RF generator and coil, torch, and injector. However, a difference between the ^40^Ar + Ca^4+^ beam intensity between the base-Neoma and Neoma MS/MS is harder to explain because the key design difference here is the pre-cell mass filter which would not prevent ^40^Ar + Ca^4+^ ions from entering the ESA due to their *m*/*z*, unless multiply charged ions are also generated in the ESA and/or in the ion path beyond.

**Fig. 8:**
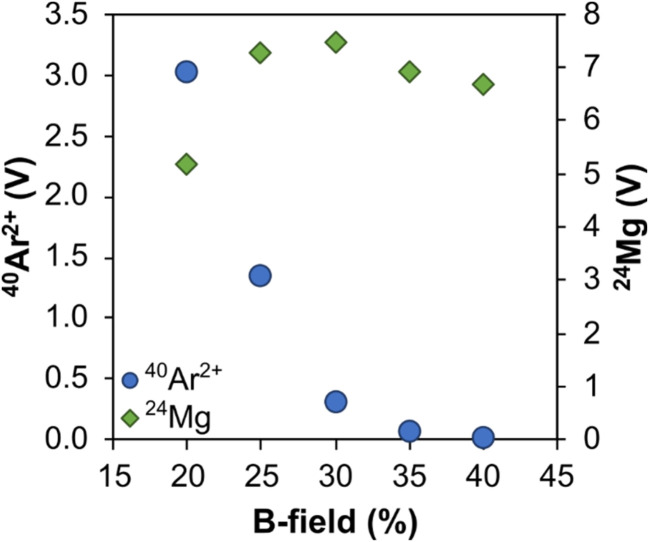
Intensity of ^40^Ar^2+^ and ^24^Mg beams when a 100 ppb Mg solution is analysed on a Neoma MS/MS MC-ICP mass spectrometer with varying pre-cell mass filter *B*-fields. Data are available in ESI Table S7.†

To investigate whether the use of a pre-cell mass filter does reduce the formation and/or subsequent detection of multiply charged ions, an experiment exploring the generation of ^40^Ar^2+^ at different *B*-fields was performed to test the hypothesis that as *B*-field increases, the pre-cell mass filter will gradually cut out a greater proportion of ^40^Ar ions. This test could not be performed directly on ^40^Ar + Ca^4+^ because, even at low *B*-fields (*e.g.* 5%), ions with a *m*/*z* of 40 are already fully cut out. Therefore, following tuning for sensitivity using a 100 ppb Mg solution (diluted from Inorganic Ventures, Inc. Mg single element standard) at varying *B*-fields (from 20% to 40% at 5% increments), the ^40^Ar^2+^ beam was measured (Fig. 8). As *B*-field increases and a greater proportion of the ^40^Ar beam is cut out, the ^40^Ar^2+^ decreases. The ^24^Mg signal remains broadly unaffected, indicating that the ^40^Ar^2+^ is not itself being cut, thus the pre-cell mass filter must influence the generation of multiply charged ions. The implication is that a sizable proportion of multiply charged ions may be formed within the ion beam transit path after the ICP interface, *i.e.* not solely in the ICP source as traditionally thought.

The ^40^Ar^4+^ and ^40^Ca^4+^ ions formed in the ion beam are created from collisions between ^40^Ar^+^, ^40^Ca^+^, and other sample ions, with charge transferred during these collisions. We propose that these interactions also transfer energy, resulting in quadruply charged ions with significantly greater or less energy than expected, and that these are the scattered ions that manifest as an elevated baseline in the boron mass range of many models of MC-ICP mass spectrometer hindering accurate measurement of boron isotope ratios. Whilst design features of the base-Neoma lower the baseline compared to its predecessor model the Neptune, the pre-cell mass filter in the Neoma MS/MS mass spectrometer acts to remove the ^40^Ar^+^ and ^40^Ca^+^ early, significantly reducing the opportunity for ^40^Ar^4+^ and ^40^Ca^4+^ to from in the ion beam transit. This results in the complete removal of the elevated baseline, paving the way for accurate boron isotope analysis by MC-ICP-MS without the use of pre-detector deflection or filtration.

### 3.3 Mass-load induced bias

**Fig. 9:**
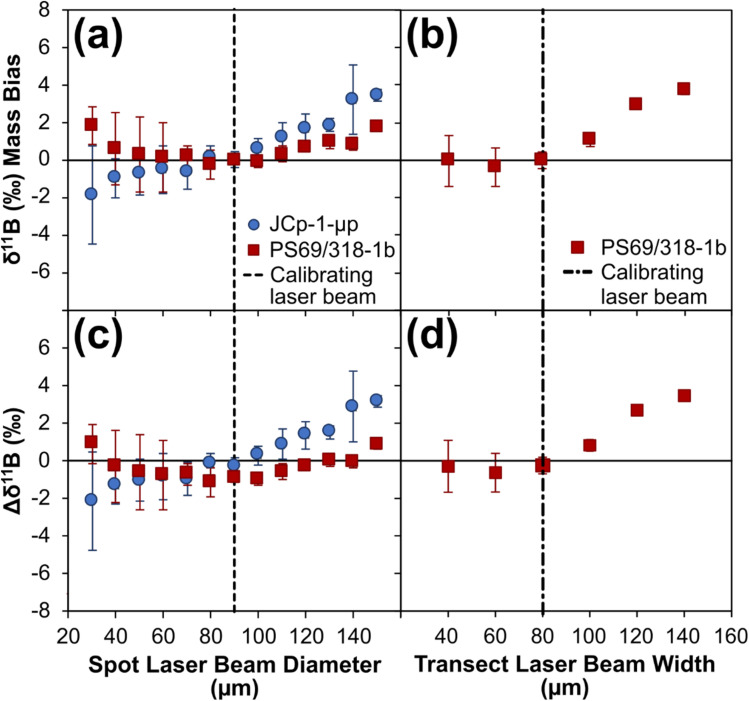
Boron isotope inaccuracy when measuring carbonate reference materials JCp-1 (pressed micropellet) and PS69/318-1b (calcitic coral fragment) under different plasma mass-loading scenarios, following normalisation to NIST SRM612. (a) and (b) Expressed as mass bias, calculated by subtracting the measured *δ*^11^B of the carbonate reference material when analysed using the same sized laser beam to that of the bracketing NIST SRM612 analyses, from the measured *δ*^11^B of the carbonate reference material when analysed using other laser beam diameters. (c) and (d) Expressed as Δ*δ*^11^B, calculated by subtracting the *δ*^11^B reference value of the carbonate reference material in question from the measured *δ*^11^B. Spot data (a and c) represent the mean of three repeat measurements (±2 SE), transect data (b and d) represent single measurements (±2 SE). Data are available in ESI Table S8.†

Inaccuracies in boron isotope ratios measured on various models of MC-ICP mass spectrometer have also been attributed to plasma mass-load induced bias.^25,30,34–36^ The use of the pre-cell mass filter to eliminate the scattered ion interference does not mitigate matrix effects arising during the laser ablation process. These are thought to relate to contrasting behaviour in terms of atomisation, ionisation, and mass fractionation within the plasma, and can manifest when samples and reference materials ablate differently due to contrasting physical properties.^37,63^ The possibility of mass-load bias being induced when using a Neoma MS/MS MC-ICP mass spectrometer was investigated firstly through the analyses of reference materials JCp-1 (pressed micropellet) and PS69/318-1b (cold water coral fragment) whilst operating the laser ablation system in spot mode (see Materials and methods). Whilst the normalising standard NIST SRM612 was ablated with a 90 μm diameter laser beam, the carbonate reference materials were ablated using a variety of laser beam sizes, with the diameter varying by 10 μm increments from 30 μm to 150 μm and thus from a 9× smaller to 2.8× larger ablation area respectively (broadly following the experiment ran by Coenen *et al.*^36^). Laser energy density and repetition rate were kept constant for all measurements (4.5 J cm^−2^ and 12 Hz respectively). Results are presented in Fig. 9a and c and ESI Table S8.†

When the analyte is sampled with a laser beam area that is smaller than that used to sample NIST SRM612, it is not possible to discern a clear mass bias relationship due to the uncertainties of the measurements (most likely because of the lower intensities being measured); all measurements are within error of 0‰ mass bias (Fig. 9a). However, when the analyte is sampled with a laser beam area that is larger than that used to sample NIST SRM612, there is a clear bias in the measured isotopic ratio which manifests as a positive relationship with the measured *δ*^11^B (Fig. 9a). Fig. 9c shows the same data plotted in terms of accuracy. This trend is the opposite to that reported by Coenen *et al.*^36^ and Sadekov *et al.*^34^ when analysing carbonates, and by Martin *et al.*^35^ and Kimura *et al.*^30^ when analysing silicates, all of which use a Neptune MC-ICP mass spectrometer. It is also counter to the predictions of Fietzke and Anagnostou^37^ who, based on their “release and diffusion” model, argue that increasing mass loads would lead to isotopically lighter values due to a lower degree of B release at the point where ions are sampled in the plasma.

This suggests that either mass-load behaviour on a Neoma MS/MS MC-ICP mass spectrometer is different to what is expected based on previous studies, or that this mass spectrometer is less sensitive to variations in mass load; ideas that future work needs to explore further. Coenen *et al.*^36^ demonstrated a clear association between mass loading and the differing ablation rates (and therefore resulting ablation depths) of analytes with contrasting physical matrices. They found that for a micropellet of JCp-1, with an ablation rate ∼2-fold larger than the NIST glass it was normalised to, the smallest degree of mass bias was achieved when the pellet was analysed with a laser spot area approximately half that of the NIST glass, *i.e.* when similar mass loads were achieved. Here, the lowest degree of mass bias is achieved when beam size is matched between analyte and standard, thus providing further evidence that mass loading is not the cause of the trends shown in Fig. 9a and c and accounting for the accuracy reported in Section 3.1.

The influence of aspect ratio on down-hole fractionation,^64^ and the aerosol size distribution arriving to the plasma^65^ that is in part controlled by laser beam size (where large beams typically generate higher proportions of larger particles^66,67^), can also induce isotopic bias, and offer alternative explanations for the bias documented here. To investigate down-hole fractionation further, a second mass-load bias test was performed by analysing reference material PS69/318-1b whilst operating the laser ablation system in transect mode (see Materials and methods). The normalising standard NIST SRM612 was ablated with an 80 by 80 μm square laser beam, and PS69/318-1b was ablated using a variety of square laser beam sizes, with the width varying by 20 μm increments from 40 by 40 μm to 140 by 140 μm. Because both PS69/318-1b and NIST SRM612 were ablated in transect mode, the ablation depths and therefore the aspect ratios were minimal and down-hole fractionation would not have been a significant factor in these measurements. Yet the data reproduces the bias recorded when operating the laser ablation system in spot mode (Fig. 9b and d): when the analyte is sampled with a laser beam area that is larger than that used to sample NIST SRM612, there is an increasing trend in the measured *δ*^11^B creating a positive isotopic mass bias; and when the analyte is sampled with a laser beam area that is smaller than that used to sample NIST SRM612, there is no clear bias and all measurements are within error of 0‰ mass bias (2 SE of ±0.6‰ or less). Down-hole fractionation is therefore unlikely to be the cause of the bias reported in Fig. 9.

By process of elimination, aerosol size distribution may therefore be the principal cause of this bias due to the known impact of laser beam size on the resulting ejecta.^66,67^ Variations in the three bias trends shown in Fig. 9 can be explained if the aerosol size distributions are also impacted by operating mode (*i.e.* spot *vs.* transect) and physical matrix (*e.g.* pressed pellets compared to mineralised materials). Further investigation into this is warranted, but irrespective of the cause, it is apparent that providing the analyte and the bracketing standard are closely matched (*i.e.* diameters no greater than ±10 μm from that of the standard), accurate measurement of boron isotope ratios is possible when coupling nanosecond laser ablation systems to the Neoma MS/MS MC-ICP mass spectrometer without matrix matching samples and reference materials. Nonetheless, it is recommended that any application of LA-MC-ICP-MS/MS for *δ*^11^B outside of the parameters explored here should carefully assess accuracy when using non-matrix matched standards because laser-induced matrix effects, such as differential aerosol generation, elemental fractionation, and plasma loading variations between samples and reference materials all remain critical challenges for LA-MC-ICP-MS approaches.

Finally, Fietzke and Anagnostou^37^ reported isotopic bias when skimmer cones are heavily coated in ablated material due to fractionation within the ICP interface. The same behaviour was observed on the Neoma MS/MS MC-ICP mass spectrometer, highlighting the need for frequent cleaning of the skimmer cone, *i.e.* between analytical sessions.

### 3.4 B/C as a tool for measuring B/Ca

On the basis that the intensity of both C and Ca should be proportional to each other in carbonate materials, it has also been investigated whether B/C ratios can be used to accurately calculate B/Ca in such materials (see ref. 6 for a similar approach). B/C is measurable because the wider mass dispersion of the Neoma detector array over that of the Neptune permits collection of ^12^C on the H5 Faraday cup when ^10^B and ^11^B are collected on the L5 and centre cups respectively. To do this, two analytical sequences were run in such a way that instrumental mass bias could also be corrected by standard-sample bracketing with JCp-1 (all other aspects of the method are as detailed in Section 2.4). Both sequences employed spot analysis, with one employing a 50 μm diameter laser beam and the other employing an 80 μm diameter laser beam. To calculate *δ*^11^B, boron isotope ratios were normalised to a JCp-1 value of 24.4‰,^49^ whilst to calculate B/Ca, the measured B/C was normalised to a JCp-1 value of 459.6, *i.e.* the reference materials B/Ca in μmol mol^−1^.^48^ Data are available in ESI Tables S9 and S10.†

**Fig. 10:**
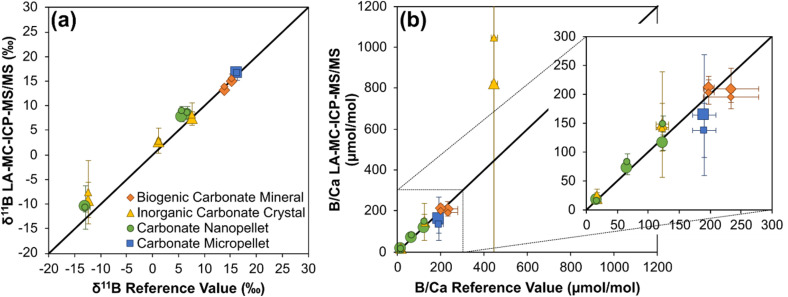
Mean *δ*^11^B (a) and B/Ca (b) ±2 SD for selected reference materials following normalisation to JCp-1. Solid black line represents 1 : 1 ratio. Data are available in ESI Tables S9 and S10.† Note that the large uncertainty on the inorganic carbonate crystals analysed is the result of small heterogeneity in B concentration of the target.

Mean accuracy of *δ*^11^B measurements, when normalised to JCp-1, was comparable to that seen when normalising to NIST SRM612, *i.e.* typically to within 1‰ of reference values (Fig. 10a). Internal precision and external reproducibility were likewise similar, highlighting that JCp-1 micropellets can also be used for mass bias correction of boron isotope ratios in carbonate.

With respect to B/Ca, measurements of the carbonate nanopellets (UWC-1-np, UWC50-np, DE-Y-np) have a mean accuracy of within 10% of reference values when analysed using an 80 μm diameter laser beam, and within 30% of reference values when analysed using a 50 μm diameter laser beam. A similar pattern is seen in the mean accuracies of biogenic carbonate minerals PS69/318-1 and PS69/318-1b, and carbonate micropellet (JCt-1), dropping from within 14% of reference values when using the larger laser beam to within 30% of reference values when using the smaller laser beam. The decline in accuracy with smaller laser beam is likely related to the quality of the ^12^C measurement, which is hindered by a large on-peak blank: typically ∼90 V. This equates to ∼50% of the total signal for the 80 μm diameter laser beam analysis compared to ∼70% of the total signal for the 50 μm diameter laser beam analysis. One avenue for improving accuracy of B/Ca measurements is therefore to target reduction of the on-peak ^12^C blank. Mean accuracy of analyses of inorganic carbonate crystals (DE-B-c, UWC-c, DE-Y-c) ranges from within 16%, to in excess of 100%, of reference values. This probably relates to heterogeneity in the boron content of these crystals, as indicated by the boron intensities recorded for these analyses. For example, 2 RSD of the ^11^B voltage is in excess of 200% when DE-B-c was analysed by both 50 μm and 80 μm diameter laser beams.

Internal precision of B/Ca analysis is typically <10% (2 RSE), irrespective of laser beam size, except when analysing the inorganic carbonate crystals where it can be as large a ∼60%. External reproducibility of carbonate nanopellets B/Ca measurements are ≤14% (2 RSD) when analysed using an 80 μm diameter laser beam and ≤17% (2 RSD) when analysed using a 50 μm diameter laser beam. External reproducibility of the biogenic carbonate minerals is of a similar order (≤17% RSD) but is much larger for both the carbonate micropellet (up to ∼60% RSD) and the inorganic carbonate crystals (sometimes in excess of 200% RSD). Both the large internal precisions and external reproducibilities of the inorganic carbonate crystals, and the large external reproducibilities of the micropellet, are likely accurate reflections of their true B/Ca variability considering: (1) the heterogeneity of the B signal in the inorganic crystals discussed above, and (2) the fact that the micropowders have not undergone further homogenisation through milling as the nanopowders have (note that JCt-1-μp measurements also document large B intensity ranges).

Based on the results presented here, laser ablation MC-ICP-MS/MS has good potential for the simultaneous characterisation of *δ*^11^B and B/Ca of carbonates when a suitable matrix-matched (in terms of bulk chemistry) is employed for the correction of instrumental mass bias. Measurements of nanopellets and organic carbonate minerals suggests that accuracy to within 10%, and both internal precisions and external reproducibilities of ∼10% or better, are achievable, which is comparable to that typically achieved by LA-ICP-MS^21,40^ and permits characterisation of typical environmental signals in a range of carbonates that often exceed 30%.^23,68,69^

## 4 Conclusions

Matrix- and mass-load induced biases and, when not employing pre-detector deflection or filtration, an interference from scattered Ca and Ar ions, typically hinders accuracy of boron isotope analysis by LA-MC-ICP-MS. However, through use of a Thermo Scientific Neoma MS/MS mass spectrometer, which combines a traditional MC-ICP mass spectrometer with a collision/reaction cell and pre-cell mass filtering technology, accuracy following mass bias correction to NIST glass standard reference materials is achieved without the use of pre-detector deflection or filtration, or the need for specialist tuning protocols. Mean values of 14 reference materials, varying in bulk chemical composition (carbonates and silicates) and boron concentration (c. 2–150 μg g^−1^), are all within uncertainty of reference values. This can be linked to: (1) design features, including a pre-cell mass filter, that prevents ^40^Ar and ^40^Ca ions from entering the ESA and successfully removes the scattered ion interference, (2) matching the laser operating parameters for samples/secondary reference materials and bracketing reference materials, and (3) limiting the mass of material being introduced into the plasma at any one time. Internal precision and external reproducibility are both primarily controlled by boron signal intensity and are typically better than 1‰ when the ^11^B intensity is at least ∼40 mV. LA-MC-ICP-MS/MS therefore offers a new opportunity for matrix independent, *in situ*, boron isotope analysis of geological materials. Furthermore, if simultaneous acquisition of B/Ca in a CaCO_3_ matrix is required, this can be determined by measuring ^12^C on the H5 Faraday cup to give a B/C ratio which will be proportional to that of the B/Ca. Based on analyses of nanopellets and carbonate biominerals, accuracy to within 10% of reference values, and both internal precisions and external reproducibility of ∼10%, is achievable.

## Data availability

The data supporting this article have been included as part of the ESI.†

## Author contributions

Christopher D. Standish: writing – review & editing, writing – original draft, visualization, validation, methodology, investigation, formal analysis, data curation, conceptualization. J. Andy Milton: writing – review & editing, resources, methodology, investigation, formal analysis, conceptualization. Rachel M. Brown: writing – review & editing, formal analysis. Gavin L. Foster: writing – review & editing, project administration, methodology, investigation, funding acquisition, conceptualization.

## Conflicts of interest

There are no conflicts to declare.

## Supplementary Material

JA-040-D5JA00028A-s001

JA-040-D5JA00028A-s002

## References

[cit1] Boron Isotopes, ed. H. Marschall and G. Foster, Springer International Publishing, Cham, 2018

[cit2] McCulloch M., Falter J., Trotter J., Montagna P. (2012). Nat. Clim. Change.

[cit3] D'Olivo J. P., McCulloch M. T., Eggins S. M., Trotter J. (2015). Biogeosciences.

[cit4] Chalk T. B., Hain M. P., Foster G. L., Rohling E. J., Sexton P. F., Badger M. P. S., Cherry S. G., Hasenfratz A. P., Haug G. H., Jaccard S. L., Martínez-García A., Pälike H., Pancost R. D., Wilson P. A. (2017). Proc. Natl. Acad. Sci. U. S. A..

[cit5] de la Vega E., Chalk T. B., Wilson P. A., Bysani R. P., Foster G. L. (2020). Sci. Rep..

[cit6] Fietzke J., Wall M. (2022). Sci. Adv..

[cit7] Standish C. D., Trend J., Kleboe J., Chalk T. B., Mahajan S., Milton J. A., Page T. M., Robinson L. F., Stewart J. A., Foster G. L. (2024). Sci. Rep..

[cit8] De Hoog J. C. M. and Savov I. P., in Boron Isotopes: the Fifth Element, ed. H. Marschall and G. Foster, Springer International Publishing, Cham, 2018, pp. 217–247

[cit9] Marschall H. R. , Advances in Isotope Geochemistry, 2018, pp. 189–215

[cit10] Hoppe P., Goswami J. N., Krähenbühl U., Marti K. (2001). Meteorit. Planet. Sci..

[cit11] Liu M.-C. and Chaussidon M., in Boron Isotopes: the Fifth Element, ed. H. Marschall and G. Foster, Springer International Publishing, Cham, 2018, pp. 273–289

[cit12] Catanzaro E. J. , Champion C. E., Garner E. L., Marinenko G., Sappenfield K. M. and Shields W. R., Boric Assay; Isotopic, and Assay Standard Reference Materials, National Bureau of Standards, 1970

[cit13] Foster G. L. , Marschall H. R. and Palmer M. R., in Boron Isotopes: the Fifth Element, ed. H. Marschall and G. Foster, Springer International Publishing, Cham, 2018, pp. 13–31

[cit14] Chen S., Littley E. F. M., Rae J. W. B., Charles C. D., Guan Y., Adkins J. F. (2023). Geochim. Cosmochim. Acta.

[cit15] Evans A. D., Standish C. D., Milton J. A., Robbins A. G., Craw D., Foster G. L., Teagle D. A. H. (2024). Geochem. Perspect. Lett..

[cit16] Vesin C., Rubatto D., Pettke T. (2024). Chem. Geol..

[cit17] Kasemann S. A., Schmidt D. N., Bijma J., Foster G. L. (2009). Chem. Geol..

[cit18] Liu M.-C., Nittler L. R., Alexander C. M. O., Lee T. (2010). Astrophys. J. Lett..

[cit19] Rollion-Bard C. and Blamart D., in Biomineralization Sourcebook, Characterization of Biominerals and Biomimetic Materials, ed. E. DiMasi and L. B. Gower, CRC Press, Boca Raton, 2014, pp. 249–262

[cit20] Fietzke J., Ragazzola F., Halfar J., Dietze H., Foster L. C., Hansteen T. H., Eisenhauer A., Steneck R. S. (2015). Proc. Natl. Acad. Sci. U. S. A..

[cit21] Chalk T. B., Standish C. D., D’Angelo C., Castillo K. D., Milton J. A., Foster G. L. (2021). Sci. Rep..

[cit22] Babila T. L., Penman D. E., Standish C. D., Doubrawa M., Bralower T. J., Robinson M. M., Self-Trail J. M., Speijer R. P., Stassen P., Foster G. L., Zachos J. C. (2022). Sci. Adv..

[cit23] MacDonald E., Foster G. L., Standish C. D., Trend J., Page T. M., Kamenos N. A. (2024). Earth Planet. Sci. Lett..

[cit24] Standish C. D., Chalk T. B., Babila T. L., Milton J. A., Palmer M. R., Foster G. L. (2019). Rapid Commun. Mass Spectrom..

[cit25] Evans D., Gerdes A., Coenen D., Marschall H. R., Müller W. (2021). J. Anal. At. Spectrom..

[cit26] Da Costa I. R., Mourão C., Récio C., Guimarães F., Antunes I. M., Ramos J. F., Barriga F. J. A. S., Palmer M. R., Milton J. A. (2014). Contrib. Mineral. Petrol..

[cit27] Míková J., Košler J., Wiedenbeck M. (2014). J. Anal. At. Spectrom..

[cit28] Yang S.-Y., Jiang S.-Y., Palmer M. R. (2015). Chem. Geol..

[cit29] le Roux P. J., Shirey S. B., Benton L., Hauri E. H., Mock T. D. (2004). Chem. Geol..

[cit30] Kimura J.-I., Chang Q., Ishikawa T., Tsujimori T. (2016). J. Anal. At. Spectrom..

[cit31] Fietzke J., Heinemann A., Taubner I., Böhm F., Erez J., Eisenhauer A. (2010). J. Anal. At. Spectrom..

[cit32] Steinhoefel G., Beck K. K., Benthien A., Richter K.-U., Schmidt-Grieb G. M., Bijma J. (2023). Rapid Commun. Mass Spectrom..

[cit33] Thil F., Blamart D., Assailly C., Lazareth C. E., Leblanc T., Butsher J., Douville E. (2016). Rapid Commun. Mass Spectrom..

[cit34] Sadekov A., Lloyd N. S., Misra S., Trotter J., D'Olivo J., Mcculloch M. (2019). J. Anal. At. Spectrom..

[cit35] Martin C., Ponzevera E., Harlow G. (2015). Chem. Geol..

[cit36] Coenen D., Evans D., Jurikova H., Dumont M., Rae J., Müller W. (2024). J. Anal. At. Spectrom..

[cit37] Fietzke J., Anagnostou E. (2023). Geostand. Geoanal. Res..

[cit38] Fietzke J., Frische M. (2016). J. Anal. At. Spectrom..

[cit39] Raitzsch M., Rollion-Bard C., Horn I., Steinhoefel G., Benthien A., Richter K.-U., Buisson M., Louvat P., Bijma J. (2020). Biogeosciences.

[cit40] Standish C. D., Chalk T. B., Saeed M., Lei F., Buckingham M. C., D'Angelo C., Wiedenmann J., Foster G. L. (2023). Geochim. Cosmochim. Acta.

[cit41] Dauphas N., Hopp T., Craig G., Zhang Z. J., Valdes M. C., Heck P. R., Charlier B. L. A., Bell E. A., Harrison T. M., Davis A. M., Dussubieux L., Williams P. R., Krawczynski M. J., Bouman C., Lloyd N. S., Tollstrup D., Schwieters J. B. (2022). J. Anal. At. Spectrom..

[cit42] Télouk P., Albalat E., Bourdon B., Albarède F., Balter V. (2023). J. Anal. At. Spectrom..

[cit43] Nie N. X., Grigoryan R., Tissot F. L. H. (2024). J. Anal. At. Spectrom..

[cit44] Télouk P., Balter V. (2024). J. Anal. At. Spectrom..

[cit45] Tanner S. D., Baranov V. I., Bandura D. R. (2002). Spectrochim. Acta, Part B.

[cit46] Huang C., Wang H., Xie L., Xu L., Wu S., Yang Y., Yang J. (2025). Spectrochim. Acta, Part B.

[cit47] Jochum K. P., Nohl U., Herwig K., Lammel E., Stoll B., Hofmann A. W. (2005). Geostand. Geoanal. Res..

[cit48] Hathorne E. C., Gagnon A., Felis T., Adkins J., Asami R., Boer W., Caillon N., Case D., Cobb K. M., Douville E., Demenocal P., Eisenhauer A., Garbe-Schönberg D., Geibert W., Goldstein S., Hughen K., Inoue M., Kawahata H., Kölling M., Cornec F. L., Linsley B. K., McGregor H. V., Montagna P., Nurhati I. S., Quinn T. M., Raddatz J., Rebaubier H., Robinson L., Sadekov A., Sherrell R., Sinclair D., Tudhope A. W., Wei G., Wong H., Wu H. C., You C.-F. (2013). Geochem. Geophys. Geosyst..

[cit49] Gutjahr M., Bordier L., Douville E., Farmer J., Foster G. L., Hathorne E. C., Hönisch B., Lemarchand D., Louvat P., McCulloch M., Noireaux J., Pallavicini N., Rae J. W. B., Rodushkin I., Roux P., Stewart J. A., Thil F., You C.-F. (2021). Geostand. Geoanal. Res..

[cit50] Foster G. L., Hönisch B., Paris G., Dwyer G. S., Rae J. W. B., Elliott T., Gaillardet J., Hemming N. G., Louvat P., Vengosh A. (2013). Chem. Geol..

[cit51] Okai T., Suzuki A., Kawahata H., Terashima S., Imai N. (2002). Geostand. Newsl..

[cit52] Inoue M., Nohara M., Okai T., Suzuki A., Kawahata H. (2004). Geostand. Geoanal. Res..

[cit53] Gerdes M. L., Valley J. W. (1994). J. Metamorph. Geol..

[cit54] Coenen D., Evans D., Hauzer H., Nambiar R., Jurikova H., Dumont M., Kanna P., Rae J., Erez J., Cotton L., Renema W., Müller W. (2024). Geochim. Cosmochim. Acta.

[cit55] Henehan M. J., Foster G. L., Rae J. W. B., Prentice K. C., Erez J., Bostock H. C., Marshall B. J., Wilson P. A. (2015). Geochem. Geophys. Geosyst..

[cit56] Trudgill M., Nuber S., Block H. E., Crumpton-Banks J., Jurikova H., Littley E., Shankle M., Xu C., Steele R. C. J., Rae J. W. B. (2024). Geochem. Geophys. Geosyst..

[cit57] Stewart J. A., Christopher S. J., Kucklick J. R., Bordier L., Chalk T. B., Dapoigny A., Douville E., Foster G. L., Gray W. R., Greenop R., Gutjahr M., Hemsing F., Henehan M. J., Holdship P., Hsieh Y., Kolevica A., Lin Y., Mawbey E. M., Rae J. W. B., Robinson L. F., Shuttleworth R., You C., Zhang S., Day R. D. (2021). Geostand. Geoanal. Res..

[cit58] Foster G. L. (2008). Earth Planet. Sci. Lett..

[cit59] Paton C., Hellstrom J., Paul B., Woodhead J., Hergt J. (2011). J. Anal. At. Spectrom..

[cit60] Breton T., Lloyd N. S., Trinquier A., Bouman C., Schwieters J. B. (2015). Procedia Earth Planet. Sci..

[cit61] Houk R. S. (1986). Anal. Chem..

[cit62] Pupyshev A. A., Semenova E. V. (2001). Spectrochim. Acta, Part B.

[cit63] Aghaei M., Lindner H., Bogaerts A. (2016). Anal. Chem..

[cit64] Horn I., Rudnick R. L., McDonough W. F. (2000). Chem. Geol..

[cit65] Fuchs J., Aghaei M., Schachel T. D., Sperling M., Bogaerts A., Karst U. (2018). Anal. Chem..

[cit66] Kroslakova I., Günther D. (2007). J. Anal. At. Spectrom..

[cit67] Kuhn B. K., Birbaum K., Luo Y., Günther D. (2010). J. Anal. At. Spectrom..

[cit68] Fallon S. J., McCulloch M. T., Alibert C. (2003). Coral Reefs.

[cit69] Babila T. L., Penman D. E., Hönisch B., Kelly D. C., Bralower T. J., Rosenthal Y., Zachos J. C. (2018). Philos. Trans. R. Soc., A.

