# Supplementary material for: Matrix independent and interference free in situ boron isotope analysis by laser ablation MC-ICP-MS/MS
Source: J Anal At Spectrom. 2025 Apr 3;40(5):1309–22. doi: 10.1039/d5ja00028a (PMC11987066; doi:10.1039/d5ja00028a)
Supplement: JA-040-D5JA00028A-s002 [file JA-040-D5JA00028A-s002.pdf]

Supplementary Information (SI) for Journal of Analytical Atomic Spectrometry.
This journal is © The Royal Society of Chemistry 2025

Supplementary Material

Matrix independent and interference free in situ boron isotope analysis by laser ablation MC-ICP-MS/MS

Christopher D. Standish[1]*, J. Andy Milton[1], Rachel M. Brown[1,2], Gavin L. Foster[1]

[1]School of Ocean & Earth Sciences, University of Southampton, National Oceanography Centre, European Way, Southampton, SO14 3ZH, UK.
[2]Aix Marseille Université, CNRS, IRD, INRAE, Coll France, CEREGE, Aix-en-Provence, France.

Iolite script for reducing B isotope data collected on the Neoma

```
# A python-based data reduction scheme for iolite 4 starts with some metadata
#/ Type: DRS
#/ Name: B_Neoma
#/ Authors: Grant Craig
#/ Description: B DRS for Neoma MSMS
#/ References: None
#/ Version: 1.0
#/ Contact: grant.craig@thermofisher.com

"""
Before functions are called a few additional objects are
added to the module:

data an interface to iolite's C++ data. E.g. you can get
existing time series data or selection groups with it
as well as make new ones.

IoLog an interface to iolite's logging facility. You can add
messages with, e.g., IoLog.debug('My message')

drs an interface to the PythonDRS C++ class in iolite from
which some built-in features can be accessed, e.g.,
baselineSubtract(group, channels, mask)

Qt imports can be done through 'iolite', e.g.
from iolite.QtGui import QLabel
"""

from iolite import QtGui
from time import sleep
import numpy as np


def runDRS():
```

```
"""
This method will be called by iolite when the user clicks
Crunch Data in the DRS window or as part of a processing
template. It should transform 'input' data into 'output'
data using the provided settings.

DRS progress can be updated via the 'message' and 'progress'
signals. These will be displayed in the iolite interface.

When finished, the 'finished' signal should be emitted.

As an example, we will do baseline subtraction of all
input channels using a DRS helper function.
"""

drs.message("Starting baseline subtract DRS...")
drs.progress(0)

# Get settings
settings = drs.settings()
print(settings)

indexChannel = data.timeSeries(settings["IndexChannel"])
rmName = settings["ReferenceMaterial"]
maskOption = settings["Mask"]
maskChannel = data.timeSeries(settings["MaskChannel"])
cutoff = settings["MaskCutoff"]
trim = settings["MaskTrim"]

# Create debug messages for the settings being used
IoLog.debug("indexChannelName = %s" % indexChannel.name)
IoLog.debug(
"Masking data = True" if maskOption else "Masking data = False")
IoLog.debug("maskChannelName = %s" % maskChannel.name)
IoLog.debug("maskCutoff = %f" % cutoff)
IoLog.debug("maskTrim = %f" % trim)

# Setup index time
drs.message("Setting up index time...")
drs.progress(5)
drs.setIndexChannel(indexChannel)

# Setup the mask
if maskOption:
drs.message("Making mask...")
drs.progress(10)
mask = drs.createMaskFromCutoff(maskChannel, cutoff, trim)
data.createTimeSeries('mask', data.Intermediate,
indexChannel.time(), mask)
else:
```

```
mask = np.ones_like(indexChannel.data())
data.createTimeSeries('mask', data.Intermediate,
indexChannel.time(), mask)

# Interp onto index time and baseline subtract
drs.message("Interpolating onto index time and baseline subtracting...")
drs.progress(25)

allInputChannels = data.timeSeriesList(data.Input)

for counter, channel in enumerate(allInputChannels):
drs.message("Baseline subtracting %s" % channel.name)
drs.progress(25 + 75*counter/len(allInputChannels))
sleep(0.5) # Sleeping only so that the progress can be observed

drs.baselineSubtract(data.selectionGroup("Baseline"), [allInputChannels[counter]], mask, 25,
100)
cps_ch = data.timeSeries(channel.name + '_CPS')
input_ch = data.timeSeries(channel.name)
cps_ch.setProperty(
'Element', input_ch.property('Element'))
cps_ch.setProperty('Mass',
input_ch.property('Mass'))

drs.message("Calculating raw ratios...")
drs.progress(50)

# Declare the channels used in the calculations:

#Be9_CPS = data.timeSeriesList(
#data.Intermediate, {'Element': 'Be', 'Mass' : '9'})[0].data()
B10_CPS = data.timeSeriesList(
data.Intermediate, {'Element': 'B', 'Mass' : '10'})[0].data()
B11_CPS = data.timeSeriesList(
data.Intermediate, {'Element': 'B', 'Mass' : '11'})[0].data()
C12_CPS = data.timeSeriesList(
data.Intermediate, {'Element': 'C', 'Mass' : '12'})[0].data()

B11_B10_Raw = B11_CPS/B10_CPS
B11_C12_Raw = B11_CPS/C12_CPS

# Gather up intermediate channels and add them as time series:
int_channel_names = ['B10_CPS', 'B11_CPS', 'C12_CPS', 'B11_B10_Raw', 'B11_C12_Raw']
int_channels = [B10_CPS, B11_CPS, C12_CPS, B11_B10_Raw, B11_C12_Raw]
for name, channel in zip(int_channel_names, int_channels):
data.createTimeSeries(name, data.Intermediate,
indexChannel.time(), channel)

drs.message("Correcting ratios...")
drs.progress(80)
```

```
StdSpline_B11_B10 = data.spline(rmName, "B11_B10_Raw").data()
try:
StdValue_B11_B10 = data.referenceMaterialData(rmName)["11B/10B"].value()
except KeyError:
IoLog.error("There was no 11B/10B value in the " + rmName +
" datafile. B DRS cannot proceed.")
drs.message("Error. See Messages")
drs.progress(100)
drs.finished()
return

print("StdSpline_B11_B10 mean = %f" % StdSpline_B11_B10.mean())
print("StdValue_B11_B10 = %f" % StdValue_B11_B10)

StdCorr_B11_B10 = (B11_B10_Raw) * StdValue_B11_B10 / StdSpline_B11_B10
data.createTimeSeries('StdCorr_B11_B10', data.Output,
indexChannel.time(), StdCorr_B11_B10)

StdSpline_B11_C12 = data.spline(rmName, "B11_C12_Raw").data()
try:
StdValue_B11_C12 = data.referenceMaterialData(rmName)["11B/12C"].value()
except KeyError:
IoLog.error("There was no 11B/12C value in the " + rmName +
" datafile. B DRS cannot proceed.")
drs.message("Error. See Messages")
drs.progress(100)
drs.finished()
return

print("StdSpline_B11_C12 mean = %f" % StdSpline_B11_C12.mean())
print("StdValue_B11_C12 = %f" % StdValue_B11_C12)

StdCorr_B11_C12 = (B11_C12_Raw) * StdValue_B11_C12 / StdSpline_B11_C12
data.createTimeSeries('StdCorr_B11_C12', data.Output,
indexChannel.time(), StdCorr_B11_C12)

StdSpline_dB11 = data.spline(rmName, "B11_B10_Raw").data()
try:
StdValue_dB11 = data.referenceMaterialData(rmName)["d11B"].value()
except KeyError:
IoLog.error("There was no d11B value in the " + rmName +
" datafile. B DRS cannot proceed.")
drs.message("Error. See Messages")
drs.progress(100)
drs.finished()
return

print("StdSpline_dB11 mean = %f" % StdSpline_dB11.mean())
print("StdValue_dB11 = %f" % StdValue_dB11)
```

```
StdCorr_dB11 = (((B11_B10_Raw / StdSpline_B11_B10) - 1)*1000) + StdValue_dB11
data.createTimeSeries('StdCorr_dB11', data.Output,
indexChannel.time(), StdCorr_dB11)

drs.message("Finished!")
drs.progress(100)
drs.finished()

def settingsWidget():
"""
This function puts together a user interface to configure the DRS.

It is important to have the last line of this function call:
drs.setSettingsWidget(widget)
"""

widget = QtGui.QWidget()
formLayout = QtGui.QFormLayout()
widget.setLayout(formLayout)

timeSeriesNames = data.timeSeriesNames(data.Input)
defaultChannelName = ""
if timeSeriesNames:
defaultChannelName = timeSeriesNames[0]

rmNames = data.selectionGroupNames(data.ReferenceMaterial)

drs.setSetting("IndexChannel", defaultChannelName)
drs.setSetting("ReferenceMaterial", "A_MAD")
drs.setSetting("Mask", False)
drs.setSetting("MaskChannel", defaultChannelName)
drs.setSetting("MaskCutoff", 0.1)
drs.setSetting("MaskTrim", 0.0)

settings = drs.settings()

indexComboBox = QtGui.QComboBox(widget)
indexComboBox.addItems(timeSeriesNames)
indexComboBox.setCurrentText(settings["IndexChannel"])
indexComboBox.currentTextChanged.connect(
lambda t: drs.setSetting("IndexChannel", t))
formLayout.addRow("Index channel", indexComboBox)

rmComboBox = QtGui.QComboBox(widget)
rmComboBox.addItems(rmNames)
if settings["ReferenceMaterial"] in rmNames:
rmComboBox.setCurrentText(settings["ReferenceMaterial"])
else:
rmComboBox.setCurrentText(rmNames[0])
```

```
drs.setSetting("ReferenceMaterial", rmNames[0])
rmComboBox.currentTextChanged.connect(lambda t: drs.setSetting("ReferenceMaterial", t))
formLayout.addRow("Reference material", rmComboBox)

verticalSpacer = QtGui.QSpacerItem(
20, 40, QtGui.QSizePolicy.Minimum, QtGui.QSizePolicy.Minimum)
formLayout.addItem(verticalSpacer)

maskCheckBox = QtGui.QCheckBox(widget)
maskCheckBox.setChecked(settings["Mask"])
maskCheckBox.toggled.connect(lambda t: drs.setSetting("Mask", bool(t)))
formLayout.addRow("Mask", maskCheckBox)

maskComboBox = QtGui.QComboBox(widget)
maskComboBox.addItems(data.timeSeriesNames(data.Input))
maskComboBox.setCurrentText(settings["MaskChannel"])
maskComboBox.currentTextChanged.connect(
lambda t: drs.setSetting("MaskChannel", t))
formLayout.addRow("Mask channel", maskComboBox)

maskLineEdit = QtGui.QLineEdit(widget)
maskLineEdit.setText(settings["MaskCutoff"])
maskLineEdit.textChanged.connect(
lambda t: drs.setSetting("MaskCutoff", float(t)))
formLayout.addRow("Mask cutoff", maskLineEdit)

maskTrimLineEdit = QtGui.QLineEdit(widget)
maskTrimLineEdit.setText(settings["MaskTrim"])
maskTrimLineEdit.textChanged.connect(
lambda t: drs.setSetting("MaskTrim", float(t)))
formLayout.addRow("Mask trim", maskTrimLineEdit)

verticalSpacer2 = QtGui.QSpacerItem(
20, 40, QtGui.QSizePolicy.Minimum, QtGui.QSizePolicy.Expanding)
formLayout.addItem(verticalSpacer2)

drs.setSettingsWidget(widget)
```
